# Effects of Exercise Training on Circulating Biomarkers of Endothelial Function in Pulmonary Arterial Hypertension

**DOI:** 10.3390/biomedicines11071822

**Published:** 2023-06-25

**Authors:** Diego A. Rodríguez-Chiaradía, Karys Khilzi, Isabel Blanco, Anna Rodó-Pin, Clara Martin-Ontiyuelo, Anna Herranz Blasco, Jessica Garcia-Lucio, Lluis Molina, Ester Marco, Esther Barreiro, Lucilla Piccari, Victor I. Peinado, Agustín R. Garcia, Olga Tura-Ceide, Joan Albert Barberà

**Affiliations:** 1 Pulmonology Department-Muscle Wasting and Cachexia in Chronic Respiratory Diseases and Lung Cancer Research Group, IMIM-Hospital del Mar, Parc de Salut Mar, Department of Medicine and Life Sciences (MELIS), Universitat Pompeu Fabra (UPF), Barcelona Biomedical Research Park (PRBB), 08003 Barcelona, Spain; kkhilzi@psmar.cat (K.K.); arodo@psmar.cat (A.R.-P.); aherranzblasco@psmar.cat (A.H.B.); ebarreiro@imim.es (E.B.); lucilla.piccari@gmail.com (L.P.); 2 Biomedical Research Networking Centre on Respiratory Diseases (CIBERES), 28029 Madrid, Spain; iblanco2@clinic.cat (I.B.); clmartin@clinic.cat (C.M.-O.); jssc5@hotmail.com (J.G.-L.); vpeinado@recerca.clinic.cat (V.I.P.); olgaturac@gmail.com (O.T.-C.); jbarbera@clinic.cat (J.A.B.); 3 Department of Pulmonary Medicine, Hospital Clínic-Institut d’Investigacions Biomèdiques August Pi i Sunyer (IDIBAPS); University of Barcelona, 08036 Barcelona, Spain; garcia83@recerca.clinic.cat; 4 Cardiology Department, IMIM-Hospital del Mar, Parc de Salut Mar, Department of Medicine and Life Sciences (MELIS), Universitat Pompeu Fabra (UPF), Barcelona Biomedical Research Park (PRBB), 08003 Barcelona, Spain; lmolina@psmar.cat; 5 Physical Medicine and Rehabilitation Department, Hospital Del Mar-Hospital de L’Esperança, Parc de Salut Mar, Rehabilitation Research Group, Institut Hospital del Mar d’Investigacions Mèdiques (IMIM), 08003 Barcelona, Spain; emarco@psmar.cat; 6 School of Medicine, Universitat Internacional de Catalunya, Sant Cugat del Vallès, 08017 Barcelona, Spain; 7 Department of Experimental Pathology, Institut d’Investigacions Biomèdiques de Barcelona (IIBB), CSIC-IDIBAPS, 08036 Barcelona, Spain; 8 Department of Pulmonary Medicine, Dr. Josep Trueta University Hospital de Girona, Santa Caterina Hospital de Salt and the Girona Biomedical Research Institut (IDIBGI), 17190 Girona, Spain

**Keywords:** pulmonary hypertension, exercise training, endothelial function

## Abstract

Introduction: In stable patients with pulmonary arterial hypertension (PAH), pulmonary rehabilitation (PR) is an effective, safe and cost-effective non-pharmacological treatment. However, the effects of PR on vascular function have been poorly explored. This study aimed to compare the amounts of circulating progenitor cells (PCs) and endothelial microvesicles (EMVs) in patients with PAH before and after 8 weeks of endurance exercise training as markers of vascular competence. Methods: A prospective study of 10 consecutive patients with PAH that successfully finished a PR program (8 weeks) was carried out before and after this intervention. Levels of circulating PCs defined as CD34+CD45low progenitor cells and levels of EMVs (CD31+ CD42b-) were measured by flow cytometry. The ratio of PCs to EMVs was taken as a measure of the balance between endothelial damage and repair capacity. Results: All patients showed training-induced increases in endurance time (mean change 287 s). After PR, the number of PCs (CD34+CD45low/total lymphocytes) was increased (*p* < 0.05). In contrast, after training, the level of EMVs (CD31+ CD42b-/total EMVs) was reduced. The ratio of PCs to EMVs was significantly higher after training (*p* < 0.05). Conclusion: Our study shows, for the first time, that endurance exercise training in patients with stable PAH has a positive effect, promoting potential mechanisms of damage/repair in favor of repair. This effect could contribute to a positive hemodynamic and clinical response.

## 1. Introduction

Pulmonary arterial hypertension (PAH) is a rare disease characterized by an abnormal rise in pressure in the pulmonary arteries followed by right ventricle overload, which can lead to death due to heart failure [1]. Despite the severity of this disease and the fact that its diagnosis and treatment are now consolidated in specialized clinical practice, the understanding of its pathogenesis still constitutes a challenge. Part of the complexity resides in its heterogeneity, since PAH may present itself in a variety of forms ranging from idiopathic to hereditary and to forms associated with other pathologies such as congenital heart disease, connective tissue disease, liver disease and the use of certain drugs and toxins. Current pharmacological treatments for PAH target altered signaling pathways in endothelial cells, but the varied response to treatment has a significant impact on survival and quality of life [1]. However, despite the long-standing lack of recommendations on exercise training, rehabilitation programs appear nowadays to be an adequate intervention in all stages of the disease [2].

Pulmonary rehabilitation (PR) is the most important non-pharmacological treatment for a wide range of respiratory and cardiovascular diseases [2]. This safe and cost-effective intervention is considered appropriate for patients with PAH according to the current guidelines [1]. In this line, several studies and clinical trials have demonstrated that PR improves aerobic capacity, decreases pulmonary vascular resistance and increases right ventricle function, leading to an improvement in quality of life [3]. Although the underlying mechanisms through which PR benefits PAH patients remains unclear, several effects on vascular [4] and right ventricular remodeling [5], inflammatory response [6], muscle function [7] and oxidative stress [8] have been identified as major improvements resulting from exercise-based interventions in PAH. Surprisingly, in patients with PAH there are few published data about the impact of PR on endothelial function [9,10,11], a recognized factor of vascular homeostasis [12]. Moreover, it has been established that endothelial dysfunction plays a critical role in the pathogenesis of many vascular diseases, including PAH [13]. Certainly, PR has been associated with improvements in endothelial function in this vascular disease, but its contribution to enhancing PAH is not entirely clear. In fact, the mechanisms by which exercise improves endothelial function are still unknown, since the evaluation of the endothelium is quite complex in the clinical scenario [14]. Circulating progenitor cells (PCs) and endothelial microvesicles (EMVs) are two promising non-invasive biomarkers, which may reveal endothelial function status and endogenous repair capacity [15].

PCs are bone-marrow-derived cells, mobilized into the circulation in response to vascular injury with the aim of maintaining and restoring normal endothelial cell function [16]. Experimental and clinical studies have shown an increase in the number of PCs after PR independently of the training program, age of the subjects and the presence of comorbidities [17].

EMVs are small vesicles (0.1–1 μm diameter) that are released from endothelial cells in response to inflammation, mechanical stress or oxidative stress at the endothelium level [15]. While healthy people preserve low levels of EMVs reflecting adequate endothelial balance, high circulating levels of EMVs have been found in several cardiovascular disorders and their levels are positively correlated with endothelial dysfunction. However, information regarding the effects of PR on these vascular biomarkers is limited [18].

Until now, no study has investigated the effects of exercise training on PCs and EMVs as non-invasive tools to evaluate the endothelium response in patients with PAH. Therefore, the current experimental study aimed to compare the levels of PCs and EMVs in patients with PAH before and after 8 weeks of endurance exercise training as markers of vascular competence.

## 2. Methods

### 2.1. Patients

**Figure 1:**
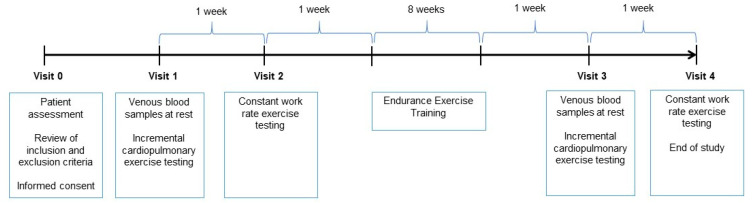
Study flow-chart.

Ten patients with stable PAH were enrolled from outpatient clinics. The study was approved by the local ethics committee (2013/5089/I) and informed consent was obtained. All patients were stable (no hospitalization or change in medication, no unplanned visits to the hospital during the previous 2 months) on disease-targeted medication for at least 8 weeks prior to inclusion. Subjects with PH not classified in Group 1 according to the classification of the 6th World Symposium on PH, left heart disease and respiratory disorders were excluded. Patients with any comorbid conditions that limited physical activity were excluded. The study flow chart is summarized in Figure 1.

### 2.2. Exercise Capacity

Exercise capacity was assessed by a cardiopulmonary exercise test. Basal condition was evaluated via breathing at rest for 3 min; after that, subjects pedaled on an electrically braked cycloergometer (CardiO2 cycle Medical Graphics Corporation, St. Paul, MN, USA and Ergoline Ergometrix 900, Uberprüfung, Germany). An integrated breath-by-breath computer system recorded cardiorespiratory variables during the test. Patients were stimulated to continue until they could no longer sustain the target pedaling frequency (55–65 rpm). Workload was increased by 10 W/min [19]. Pulmonary gas exchange and ventilatory data were obtained from calibrated signals derived from response gas analyzers and a mass flow sensor. The recorded variables during each respiration were pulmonary oxygen uptake (VO2), pulmonary carbon dioxide output (VCO2), respiratory exchange ratio, minute ventilation (VE) and tidal volume and respiratory rate (RR). Heart rate (HR) was evaluated using a three-lead online electrocardiogram and oxygen saturation by pulse oximetry (SpO2). A ventilatory limitation was calculated as the ratio of peak VE to the estimated maximal ventilatory capacity (MVC; (peak V’E/MVC) × 100). A constant work rate exercise (CWRET) test involving pedaling at 75% of peak work rate (Wpeak) until exhaustion was carried out before and after the exercise training program to evaluate endurance time [20].

### 2.3. Endurance Exercise Training

All patients exercised on a cycle ergometer (Ergoline; Wüerzburg, Germany) for 24 sessions for 8 weeks (1 h per session, 3 days/week). After a 5 min warm-up of cycling at 20% Wpeak, patients performed exercise consisting of 40 min of cycling as continuous training at 60% Wpeak, followed by a recovery period of 5 min cycling at 20% Wpeak. The rate of pedaling during these training sessions was kept at 60–70 rpm. The progress of the work rate during each training period was also decided on an individual basis, according to patients’ symptoms, thereby maximizing the training effect. In all cases, pulse oximetry and heart rate were continuously monitored (Pulsox-300i, Konica Minolta, Osaka, Japan) during the training sessions.

As recommended by the current statement, the primary outcome was the achievement of at least a 100 s improvement from pre-exercise training in the endurance time (ET) obtained during a constant work rate exercise test (CWRET) using the cycle ergometer [20,21].

### 2.4. Blood Sampling

Venous blood samples were obtained at rest before cardiopulmonary exercise and before and after the 8-week endurance exercise training; they were obtained by peripheral venipuncture and placed into two 4.5 mL sodium citrate tubes (Becton Dickinson, Plymouth, UK) to measure circulating EVs, and into two 4.5 mL tubes with EDTA (Becton Dickinson, Plymouth, UK) to measure circulating PCs.

### 2.5. Assessment of Circulating Endothelial Microparticles

To assess circulating EMVs, flow cytometry was used to determine the expression of the platelet endothelium adhesion molecule (PECAM-1, CD31) in the absence of the platelet-specific glycoprotein marker CD42b, as previously described by our team [22]. First, peripheral blood was collected and centrifuged for 10 min (800× *g*, 4 °C) within one hour of collection to prepare platelet-rich plasma (PPP). The supernatant was then centrifuged for 10 min (300× *g*, 23 °C) within five minutes to obtain platelet-poor plasma (PPP) and discard any cell debris. EMV phenotype analysis was conducted based on size and fluorescence, identifying events less than 1 µm in forward (size) and side (density) light scatter plots using size calibration microspheres (FluoSpherescarboxylate-modified microspheres 1.0 mm, yellow-green fluorescent (505/515), Invitrogen, Oregon, USA). EMV levels were evaluated by comparing them with calibrator beads (Perfect Count Microspheres Cytognos, Salamanca, Spain) with a known concentration, using 2000 event beads (PE) as a stop time.

**Figure 2:**
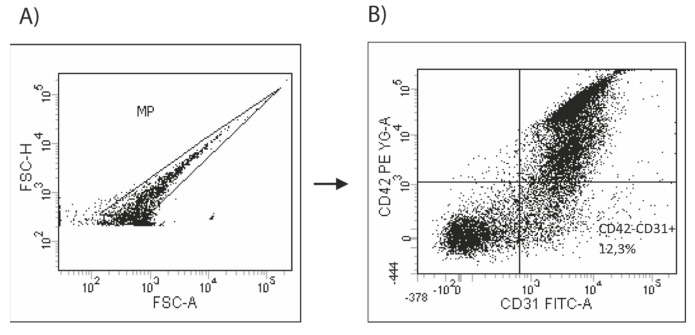
Gating strategy for endothelial microparticles (EMVs). (**A**) MPs analysis based on size and fluorescence; (**B**) sample analyzed by two-color fluorescence histograms as CD31+CD42b- (total EMVs).

To analyze different cell types, fluorescent labeling techniques were employed. For the negative control, fluorescence-minus-one (FMO) tubes were used (100,000 MPs/mL), as well as each phenotype (500,000 MPs/mL), and they were stained for 45 min at room temperature with pre-conjugated anti-human monoclonal antibodies and isotype controls: anti-CD31-FITC, anti-CD42b-PE and anti-IgG1k-PE isotype controls (BD PharmigenTM, San Jose, CA, USA). To provide negative controls, the fluorescence-minus-one technique was employed [23]. The samples were lysed using two- or three-color fluorescence histograms and labeled as CD31+CD42b- microparticles. Compensation assessment was performed using single-antibody conjugates and compensation fluorochrome beads. The samples were acquired using LRSFortessa flow cytometrywith a bandpass filter of 530 nm (FITC) and 585 nm (PE/PI). We acquired 100,000 MPs/events, and the data were analyzed using FACSDIVA from Tree Star, OR. We show an example of how the analysis was performed below (Figure 2).

### 2.6. Assessment of Circulating Progenitor Cells

**Figure 3:**
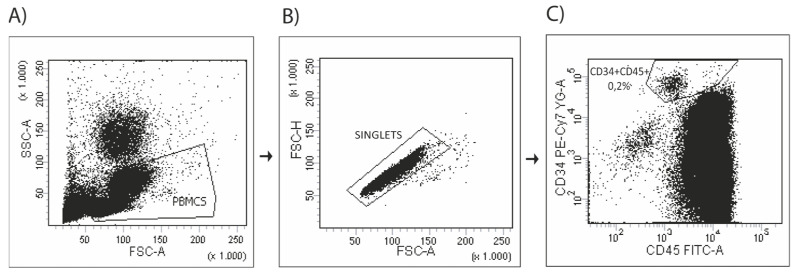
Gating strategy for progenitor cells (PCS). (**A**) Peripheral blood mononuclearcells (PBM C) selection based on forward and side, (**B**) Singlet selection withno aggregates; (**C**) Sample analyzed by two-color fluorescence histograms asCD34+CD45low cells.

The number of circulating progenitor cells was evaluated by flow cytometry using antibodies against CD45 (pan-leukocyte marker), CD133 (sub-population of hematopoietic stem cells) and CD34 (mature and progenitor endothelial cells) as previously described [22]. In brief, circulating PCs were isolated from fresh peripheral blood by Ficoll density gradient centrifugation, washed once with phosphate-buffered saline (PBS) supplemented with 2% of fetal calf serum (FCS) and resuspended at 2 × 10^6^ cells for FMO tubes and at 4 × 10^6^ cells for sample tubes. Circulating PCs were stained and analyzed by flow cytometry for phenotypic expression of surface markers using pre-conjugated anti-human monoclonal antibodies and isotype controls anti-CD45-FITC (BD Pharmingen TM, San Jose, CA, USA), anti-CD34-PECy7 (eBiosciences, San Diego, CA, USA), anti-CD133-PE (MACS Miltenyi Biotec, Bergisch Gladbach, Germany), anti-IgG1k-PECy7 isotype control (eBiosciences, San Diego, CA, USA), anti-IgG1k-FITC isotype control (BD PharmingenTM, San Jose, CA) and anti-IgG1k-PE isotype control (BD PharmingenTM, San Jose, CA, USA). The fluorescence-minus-one technique was employed to provide negative controls [24]. After 45 min of incubation, cells were washed and resuspended in 500 μL of PBS + 2% FCS, and then we proceeded to flow cytometry analysis. A total of 750,000 CD45^+^ events were run through the LRSFortessa (BD Bioscience, San Jose, CA, USA). The data were analyzed using FACSDIVA (Tree Star, OR, USA) following the ISHAGE (International Society of Hematotherapy and Graft Engineering) gating strategy previously published [25]. We show an example of how the analysis was performed below (Figure 3).

### 2.7. Statistical Analysis

Data are shown as mean ± standard deviation (SD). The groups were compared using t student test or one-way ANOVA and post hoc pairwise comparisons using the Student–Newman–Keuls test for normally distributed variables, or Mann–Whitney Rank Sum Test and Kruskal–Wallis one-way analysis of variance on ranks test for non-normally distributed variables. Pre- and post-training comparisons were assessed by paired t-test for normally distributed variables and Signed Test for non-normally distributed variables. Statistical significance was considered at a *p* value < 0.05.

## 3. Results

### 3.1. Study Population

Ten consecutive patients with stable PAH under optimized medication therapy were included. Patients were treated with calcium channel blockers (*n* = 1 with nifedipine), phosphodiesterase-5 inhibitors (*n* = 10; 7 with tadalafil and 3 with sildenafil), endothelin receptor antagonists (*n* = 9; 5 with ambrisentan, 3 with macitentan and 1 with bosentan) or prostanoids (*n* = 1 with iloprost). The clinical, functional and hemodynamic characteristics of patients are shown in Table 1. Patients had moderate to severe PAH and the majority were in functional class II with combined therapy at the moment of inclusion.

### 3.2. Baseline Profiles

**Figure 4:**
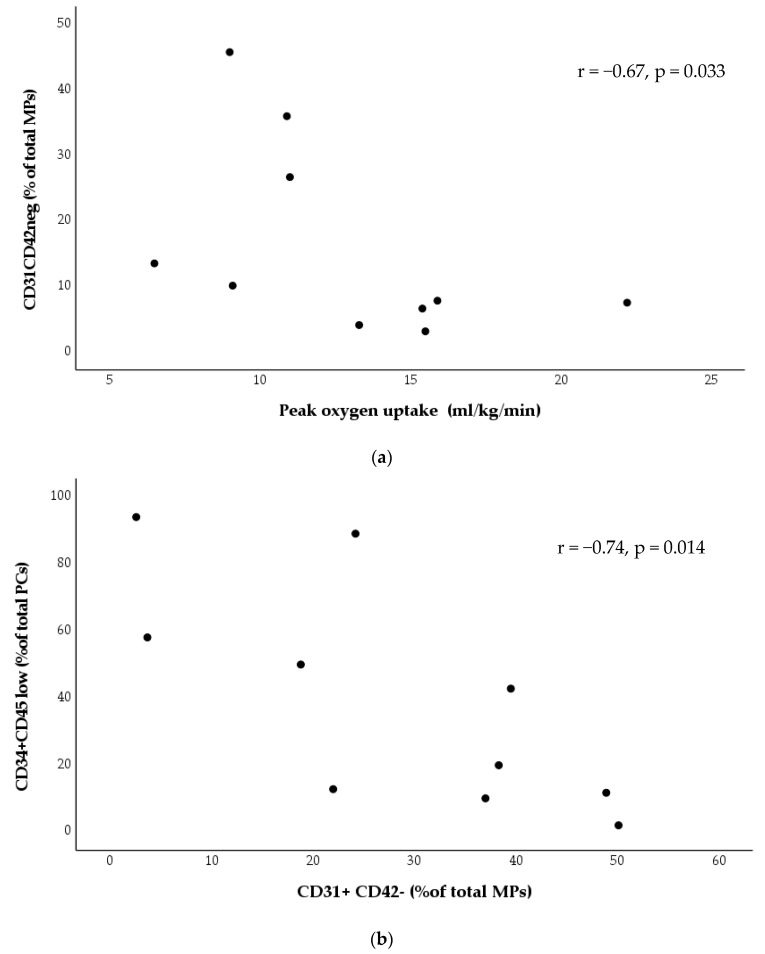
(**a**) Scatter plot representation of correlations between the number of circulating EMVs (CD31CD42neg) and peak oxygen uptake (mL/kg/min) before exercise training; (**b**) scatter plot representation of correlation between the number of circulating EMVs and PCs before exercise training.

Before exercise training, there was significant inverse correlation between the number of circulating EMVs (CD31, CD42neg) and peak oxygen uptake (Figure 4a). Also, there was a significant inverse correlation between the number of circulating EMVs and PCs (Figure 4b).

### 3.3. Effects of Exercise Training

After 8 weeks of exercise training, all patients showed training-induced increases in endurance time (mean increase relative to baseline +287 s). The complete data about physiological effects due to exercise training are presented in Table 2.

**Figure 5:**
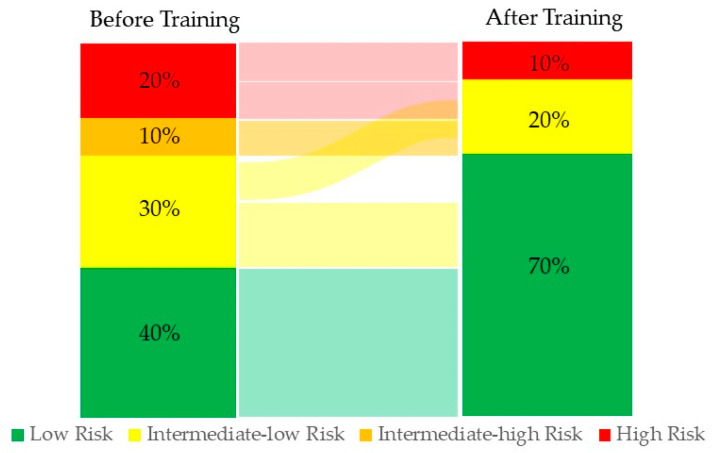
Change in risk from baseline to follow-up is shown for the four-stratum model. Risk at baseline (before training) and follow-up (after training).

Changes in the mortality risk were observed in the group after applying the four-stratum model [1] before and after the exercise training program (Figure 5).

**Figure 6:**
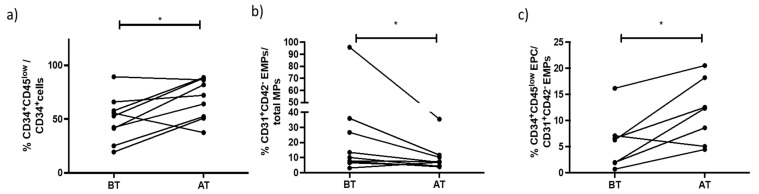
Patients with PAH after an exercise training program showed higher numbers of progenitor cells (PC) defined as CD34^+^CD45^low^ (* *p* = 0.01) (**a**); lower numbers of endothelial microparticles defined as CD31+CD42b- (* *p* = 0.02) (**b**); and increased ratio PCs/EMVs (* *p* = 0.03) (**c**) compared to before training program. BT = Before Training. AT = After Training.

After exercise training, the number of PCs (CD34+CD45low/total lymphocytes) increased (*p* < 0.05), whereas those of EMVs (CD31+ CD42b-/total MVs) decreased. The ratio of PCs to EMVs was significantly higher after training (*p* < 0.05) (Figure 6).

## 4. Discussion

The present study investigated the modifications in endothelial vascular biomarkers in PAH patients induced by 8 weeks of endurance exercise training. Our major findings were that, after this exercise program, the percentage of circulating PCs was markedly increased with a significant reduction in the amount of circulating EMVs. Importantly, as a consequence of exercise training, the ratio of PCs to EMVs was increased, contributing to improved endothelial homeostasis.

Our result is in accordance with previous studies that have shown the beneficial effect of exercise training with significant improvements in circulating PCs in chronic complex diseases associated with endothelial dysfunction [26,27]. Although no studies have assessed the effect of exercise training on circulating PCs in patients with PAH, a favorable increase in circulating PCs has been related with positive effects in patients under PAH treatment [3]. Therefore, we can postulate that an increase in circulating PCs is related to a restoration of the endothelial balance at the pulmonary vascular level and contributes to an improvement of vascular remodeling [28]. This hypothesis is supported by previous animal [29] and clinical studies [30] that showed a significant increase in cardiac output after a training program, demonstrating a direct influence on right ventricular function and pulmonary vascular disease progression. In line with our results, further studies indicate an inverse correlation between exercise capacity and PCs [24] before endurance training, and this correlation may be modulated after the program with potential implications for vascular remodeling mechanisms in these patients.

Regarding the angiogenic processes, EMVs play a key role as a marker of endothelial health. EMVs [23] are complex vesicular structures shed from activated or apoptotic endothelial cells. Although few studies have investigated EMVs after endurance exercise in healthy subjects [31] or patients [18], present evidence suggests that exercise training may reduce the pathophysiological potential of circulating EMVs. While low concentrations of EMVs have been reported as proangiogenic [32], high concentrations of EMVs have been described as anti-angiogenic, as they decrease the formation of capillary-like structure through the production of reactive oxygen species [33]. Importantly, we found that a reduction in the number of EMVs was associated with improved exercise capacity after 8 weeks of endurance training in patients with PAH. These findings are in accordance with previous data, showing a decrease in EMVs, suggesting reduced vascular damage and vesiculation at rest after the training period [34].

In the current study, we did find a relationship between circulating EMVs and PCs only before exercise training, which reflects the complex interactions between both biomarkers of endothelial function modulated by chronic exercise [16].

The EMVs to PCs ratio is a measure of the balance between endothelial damage and repair capacity [22]. In our patients, the PC to EMVs ratio was increased after exercise training, indicating for the first time the impact of exercise training on restoring the balance between endothelial damage and repair capacity in PAH patients, as previously demonstrated in other diseases with endothelial dysfunction [28]. In this line, our work reinforces the current recommendations regarding the need to include these patients in rehabilitation programs and suggests an impact on four strata of risk stratification assessment (Figure 5).

The major limitation of the present study is the small sample size, limiting our ability to draw solid conclusions; however, this is a first evaluation of the effects of exercise training on specific endothelial vascular biomarkers, increasing our knowledge of underlying mechanisms that may improve exercise tolerance in patients with PAH after rehabilitation programs. Moreover, PR seemed to have a protective effect in these patients, considering the observed increase in PCs. Another limitation is the fact that the patients had different types of PAH; however, subgroups of patients within PAH are largely considered to share pathophysiological mechanisms and molecular alterations, which may explain why they would respond similarly to PR. Further studies will answer several unsolved questions, such as the potential underlying molecular mechanisms in different subtypes of PAH and the long-term benefits.

## 5. Conclusions

Our study shows that endurance exercise training in patients with stable PAH patients has a positive effect, promoting potential mechanisms of damage/repair to favor repair. This effect could contribute to the improvement of pulmonary hemodynamics and of clinical response.

## Figures and Tables

**Table 1:** Basal characteristics of the study population.

Age (years)	54 (12.6)
Male (%)	20
Body Mass Index (kg/m^2^)	26.5 (10)
Mean Pulmonary Arterial Pressure (mmHg)	39.4 (8.6)
Pulmonary Vascular Resistance (Wood unit)	6.8 (3)
Cardiac Index (L/m^2^)	2.5 (0.6)
Pulmonary Arterial Wedge Pressure (mmHg)	9.5 (4)
Right Atrial Pressure (mmHg)	8 (3.8)
FEV_1_/FVC (% pred)	69.4 (7)
FEV_1_ (% pred)	68 (6.8)
FVC (% pred)	77 (9)
DL_CO_ (% pred)	63 (20)
TLC (% pred)	98 (15)
FC II (%)	90
Double therapy (%)	80
**GROUP (*n*)**	
CHD	2
SS	2
HIV	2
Idiopathic	3
SLE	1

Data are presented as mean (SD) or number (percentage). FEV_1_: forced expiratory volume in 1 s; FVC: forced vital capacity; TLC: total lung capacity; DL_CO_: diffusing capacity of the lung for carbon monoxide; TLC: total lung capacity; FC: functional class; CHD: congenital heart disease; SS: systemic sclerosis; HIV: human immunodeficiency virus; SLE: systemic lupus erythematosus.

**Table 2:** Basal characteristics in the incremental cardiopulmonary exercise testing (ICPET) and effects of pulmonary rehabilitation on constant work rate exercise testing (CWRET).

Exercise Parameters	Basal		
**ICPET**			
Workload (Watts) peak	59 (29)		
Workload (% predicted) peak	55 (16)		
VO_2_ (mL/kg/min) peak	13 (4)		
VO_2_ (% predicted) peak	50 (14)		
VO_2_ (% predicted) AT	28 (17)		
V_E_ (% max) peak	60 (16)		
HR peak (beat per minute)	126 (19)		
HR (% predicted) peak	76 (12)		
O_2_ pulse peak	6.3 (2)		
V_E_/VCO_2_ AT	35 (2)		
Borg dyspnea final	6 (3)		
Borg leg final	5 (2)		
**CWRET**	Before ET	After ET	*p* value
Endurance time (seconds)	260 (125)	527 (299)	0.004
VO_2_ (isotime) (L/min)	0.61 (0.1)	0.75 (0.2)	0.042
V_E_ (isotime)	36 (11)	35 (11)	0.534
HR (isotime)	132 (15)	122 (13)	0.042
O2 pulse (isotime)	4 (0.5)	6 (1)	0.038

Data are presented as mean (SD) and number (percentage). Peak V’O_2_, peak oxygen uptake; V’_E_ max, maximum minute ventilation; HR, heart rate; V’E/V’CO_2_, ventilatory equivalent for carbon dioxide.

## Data Availability

The data that support the findings of this study are available from the corresponding author upon reasonable request.

## Abbreviations

PAH	Pulmonary arterial hypertension
PR	Pulmonary rehabilitation
PC	Circulating progenitor cells
EMV	Endothelial microvesicles
VO_2_	Oxygen uptake pulmonary
VCO_2_	Carbon dioxide output
RER	Respiratory exchange ratio
VE	Minute ventilation respiratory rate
RR	Respiratory rate
HR	Heart rate
SpO_2_	Oxygen saturation by pulse oximetry
CWRET	Constant work rate exercise testing
Wpeak	Peak work rate
ET	Endurance time

## References

[1] Humbert M., Kovacs G., Hoeper M.M., Badagliacca R., Berger R.M.F., Brida M., Rosenkranz S. (2022). 2022 ESC/ERS Guidelines for the diagnosis and treatment of pulmonary hypertension. Eur. Heart J..

[2] Spruit M.A., Singh S.J., Garvey C., ZuWallack R., Nici L., Rochester C., Hill K., Holland A.E., Lareau S.C., Man W.D.-C. (2013). An Official American Thoracic Society/European Respiratory Society Statement: Key Concepts and Advances in Pulmonary Rehabilitation. Am. J. Respir. Crit. Care Med..

[3] Grünig E., Eichstaedt C., Barberà J.-A., Benjamin N., Blanco I., Bossone E., Cittadini A., Coghlan G., Corris P., D’Alto M. (2019). ERS statement on exercise training and rehabilitation in patients with severe chronic pulmonary hypertension. Eur. Respir. J..

[4] Colombo R., Siqueira R., Becker C.U., Fernandes T.G., Pires K.M., Valença S.S., Souza-Rabbo M.P., Araujo A.S., Belló-Klein A. (2013). Effects of exercise on monocrotaline-induced changes in right heart function and pulmonary artery remodeling in rats. Can. J. Physiol. Pharmacol..

[5] Soares L.L., Drummond F.R., Rezende L.M.T., Costa A.J.L.D., Leal T.F., Fidelis M.R., Neves M.M., Prímola-Gomes T.N., Carneiro-Junior M.A., Reis E.C.C. (2019). Voluntary running counteracts right ventricular adverse remodeling and myocyte contraction impairment in pulmonary arterial hypertension model. Life Sci..

[6] Harbaum L., Renk E., Yousef S., Glatzel A., Lüneburg N., Hennigs J.K., Oqueka T., Baumann H.J., Atanackovic D., Grünig E. (2016). Acute effects of exercise on the inflammatory state in patients with idiopathic pulmonary arterial hypertension. BMC Pulm. Med..

[7] Mainguy V., Maltais F., Saey D., Gagnon P., Martel S., Simon M., Provencher S. (2010). Effects of a Rehabilitation Program on Skeletal Muscle Function in Idiopathic Pulmonary Arterial Hypertension. J. Cardiopulm. Rehabil. Prev..

[8] Silva J.M.A., Tucci P.J.F., Conzatti A., de Lima Seolin B.G., Fernandes T.R.G., da Rosa Araújo A.S., Belló-Klein A. (2016). Exercise training contributes to H2O2/VEGF signaling in the lung of rats with monocrotaline-induced pulmonary hypertension. Vasc. Pharm..

[9] Moreira-Gonçalves D., Ferreira R., Fonseca H., Padrão A.I., Moreno N., Silva A.F., Vasques-Nóvoa F., Gonçalves N., Vieira S., Santos M. (2015). Cardioprotective effects of early and late aerobic exercise training in experimental pulmonary arterial hypertension. Basic Res. Cardiol..

[10] Zimmer A., Teixeira R.B., Bonetto J.H.P., Siqueira R., Carraro C.C., Donatti L.M., Hickmann A., Litvin I.E., Godoy A.E.G., Araujo A.S. (2017). Effects of aerobic exercise training on metabolism of nitric oxide and endothelin-1 in lung parenchyma of rats with pulmonary arterial hypertension. Mol. Cell Biochem..

[11] Potus F., Malenfant S., Graydon C., Mainguy V., Tremblay È., Breuils-Bonnet S., Ribeiro F., Porlier A., Maltais F., Bonnet S. (2014). Impaired Angiogenesis and Peripheral Muscle Microcirculation Loss Contribute to Exercise Intolerance in Pulmonary Arterial Hypertension. Am. J. Respir. Crit. Care Med..

[12] Sandoo A., Veldhuijzen van Zanten J.J.C., Metsios G.S., Carroll D., Kitas G.D. (2015). The Endothelium and Its Role in Regulating Vascular Tone. Open Cardiovasc. Med. J..

[13] Pober J.S., Min W., Bradley J.R. (2009). Mechanisms of endothelial dysfunction, injury, and death. Annual Review of Pathology: Mechanisms of Disease. Annu. Rev. Pathol..

[14] Huertas A., Guignabert C., Barberà J.A., Bärtsch P., Bhattacharya J., Bhattacharya S., Wilkins M.R. (2018). Pulmonary vascular endothelium: The orchestra conductor in respiratory diseases Highlights from basic research to therapy. Eur. Respir. J..

[15] Sabatier F., Camoin-Jau L., Anfosso F., Sampol J., Dignat-George F. (2009). Circulating endothelial cells, microparticles and progenitors: Key players towards the definition of vascular competence. J. Cell Mol. Med..

[16] Burger D., Touyz R.M. (2012). Cellular biomarkers of endothelial health: Microparticles, endothelial progenitor cells, and circulating endothelial cells. J. Am. Soc. Hypertens..

[17] Koutroumpi M. (2012). Circulating endothelial and progenitor cells: Evidence from acute and long-term exercise effects. World J. Cardiol..

[18] Highton P.J., Martin N., Smith A.C., Burton J., Bishop N.C. (2018). Microparticles and Exercise in Clinical Populations. Exerc. Immunol. Rev..

[19] Radtke T., Crook S., Kaltsakas G., Louvaris Z., Berton D., Urquhart D.S., Kampouras A., Rabinovich R.A., Verges S., Kontopidis D. (2019). ERS statement on standardisation of cardiopulmonary exercise testing in chronic lung diseases. Eur. Respir. Rev..

[20] Puente-Maestu L., Palange P., Casaburi R., Laveneziana P., Maltais F., Neder J.A., O’Donnell D.E., Onorati P., Porszasz J., Rabinovich R. (2016). Use of exercise testing in the evaluation of interventional efficacy: An official ERS statement. Eur. Respir. J..

[21] Maltais F., Celli B., Casaburi R., Porszasz J., Jarreta D., Seoane B., Caracta C. (2011). Aclidinium bromide improves exercise endurance and lung hyperinflation in patients with moderate to severe COPD. Respir. Med..

[22] García-Lucio J., Peinado V.I., de Jover L., del Pozo R., Blanco I., Bonjoch C., Paul T. (2018). Imbalance between endothelial damage and repair capacity in chronic obstructive pulmonary disease. PLoS ONE.

[23] Tura-Ceide O., Blanco I., Garcia-Lucio J., del Pozo R., García A.R., Ferrer E., Crespo I., Rodríguez-Chiaradia D.A., Simeon-Aznar C.P., López-Meseguer M. (2021). Circulating Cell Biomarkers in Pulmonary Arterial Hypertension: Relationship with Clinical Heterogeneity and Therapeutic Response. Cells.

[24] Feher K., Kirsch J., Radbruch A., Chang H.-D., Kaiser T. (2014). Cell population identification using fluorescence-minus-one controls with a one-class classifying algorithm. Bioinformatics.

[25] Schmidt-Lucke C., Fichtlscherer S., Aicher A., Tschöpe C., Schultheiss H.-P., Zeiher A.M., Dimmeler S. (2010). Quantification of Circulating Endothelial Progenitor Cells Using the Modified ISHAGE Protocol. PLoS ONE.

[26] Werner N., Kosiol S., Schiegl T., Ahlers P., Walenta K., Link A., Böhm M., Nickenig G. (2005). Circulating Endothelial Progenitor Cells and Cardiovascular Outcomes. N. Engl. J. Med..

[27] Tahhan A.S., Hammadah M., Sandesara P.B., Hayek S.S., Kalogeropoulos A.P., Alkhoder A., Kelli H.M., Topel M., Ghasemzadeh N., Chivukula K. (2017). Progenitor Cells and Clinical Outcomes in Patients with Heart Failure. Circ. Hear Fail..

[28] Sandri M., Viehmann M., Adams V., Rabald K., Mangner N., Höllriegel R., Lurz P., Erbs S., Linke A., Kirsch K. (2015). Chronic heart failure and aging—effects of exercise training on endothelial function and mechanisms of endothelial regeneration: Results from the Leipzig Exercise Intervention in Chronic heart failure and Aging (LEICA) study. Eur. J. Prev. Cardiol..

[29] Handoko M., Man F.H.-D., Happé C., Schalij I., Musters R., Westerhof N., Postmus P., Paulus W., van der Laarse W., Vonk-Noordegraaf A. (2009). Opposite Effects of Training in Rats with Stable and Progressive Pulmonary Hypertension. Circulation.

[30] Ehlken N., Lichtblau M., Klose H., Weidenhammer J., Fischer C., Nechwatal R., Uiker S., Halank M., Olsson K., Seeger W. (2016). Exercise training improves peak oxygen consumption and haemodynamics in patients with severe pulmonary arterial hypertension and inoperable chronic thrombo-embolic pulmonary hypertension: A prospective, randomized, controlled trial. Eur. Heart J..

[31] Lansford K.A., Shill D.D., Dicks A.B., Marshburn M.P., Southern W.M., Jenkins N.T. (2016). Effect of acute exercise on circulating angiogenic cell and microparticle populations. Exp. Physiol..

[32] Burnier L., Fontana P., Kwak B.R., Angelillo-Scherrer A. (2009). Cell-derived microparticles in haemostasis and vascular medicine. Thromb. Haemost..

[33] Mezentsev A., Merks R.M., O’Riordan E., Chen J., Mendelev N., Goligorsky M.S., Brodsky S.V. (2005). Endothelial microparticles affect angiogenesis in vitro: Role of oxidative stress. Am. J. Physiol. Heart Circ. Physiol..

[34] Wilhelm E.N., Mourot L., Rakobowchuk M. (2018). Exercise-Derived Microvesicles: A Review of the Literature. Sports Med..

